# Pharmacological treatment of comorbid posttraumatic stress disorder in patients with bipolar disorder

**DOI:** 10.1192/j.eurpsy.2022.1030

**Published:** 2022-09-01

**Authors:** S. Hendriks, P. Goossens

**Affiliations:** Dimence Group Center for Mental Health Care, Specialized Center Bipolar Disorders, Deventer, Netherlands

**Keywords:** Treatment, posttraumatic stress disorder, Pharmacotherapy, bipolar disorder

## Abstract

**Introduction:**

The lifetime prevalence of comorbid posttraumatic stress disorder (PTSD) in patients with bipolar disorder (BD) is approximately 20%. Guidelines for BD give adequate pharmacological treatment options when there is a ‘pure’ bipolar disorder but lack of treatment options when there is a comorbid disorder present.

**Objectives:**

The present study aimed to review the pharmacological treatment options for comorbid PTSD in patients with BD.

**Methods:**

Literature research was conducted via PubMed, Embase and the Cochrane Library. Search terms included ‘bipolar disorder’, ‘posttraumatic stress disorder’, ‘PTSD’, ‘pharmacotherapy’ and ‘treatment’. Relevant studies were reviewed.

**Results:**

No randomized controlled trials have been conducted in patients with bipolar disorder and comorbid PTSD. Most studies included open-label studies and case-reports. No convincing scientific evidence for pharmacological treatment of comorbid PTSD in patients with BD was found. Selective serotonin reuptake inhibitors (SSRIs) are effective in the treatment of PTSD. However, SSRIs or other antidepressants are complicated due to potential induction of a manic episode or promote rapid cycling. Nevertheless, it is important to treat the bipolar patient with a mood stabilizer first before antidepressants are prescribed.

**Conclusions:**

The findings of this study show that there is no convincing scientific evidence for the pharmacological treatment of comorbid PTSD in patients with bipolar disorder. Therefore, psychotherapy is preferable. When psychotherapy is not effective, pharmacotherapy can be considered. However, randomized controlled trials are needed to obtain scientific evidence for pharmacological treatment options.

**Disclosure:**

No significant relationships.

